# Exploring Ligand Binding Domain Dynamics in the NRs Superfamily

**DOI:** 10.3390/ijms23158732

**Published:** 2022-08-05

**Authors:** Giulia D’Arrigo, Ida Autiero, Eleonora Gianquinto, Lydia Siragusa, Massimo Baroni, Gabriele Cruciani, Francesca Spyrakis

**Affiliations:** 1 Department of Drug Science and Technology, University of Turin, Via Giuria 9, 10125 Turin, Italy; 2 Molecular Horizon Srl, Via Montelino 30, 06084 Bettona, Italy; 3 National Research Council, Institute of Biostructures and Bioimaging, 80138 Naples, Italy; 4 Molecular Discovery Ltd., Theobald Street, Elstree Borehamwood, Hertfordshire WD6 4PJ, UK; 5 Department of Chemistry, Biology and Biotechnology, University of Perugia, Via Elce di Sotto 8, 06123 Perugia, Italy; 6 Consortium for Computational Molecular and Materials Sciences (CMS), Via Elce di Sotto 8, 06123 Perugia, Italy

**Keywords:** nuclear receptors, flexibility, drug design, agonists, antagonists, ligand binding domain

## Abstract

Nuclear receptors (NRs) are transcription factors that play an important role in multiple diseases, such as cancer, inflammation, and metabolic disorders. They share a common structural organization composed of five domains, of which the ligand-binding domain (LBD) can adopt different conformations in response to substrate, agonist, and antagonist binding, leading to distinct transcription effects. A key feature of NRs is, indeed, their intrinsic dynamics that make them a challenging target in drug discovery. This work aims to provide a meaningful investigation of NR structural variability to outline a dynamic profile for each of them. To do that, we propose a methodology based on the computation and comparison of protein cavities among the crystallographic structures of NR LBDs. First, pockets were detected with the FLAP*site* algorithm and then an “all against all” approach was applied by comparing each pair of pockets within the same sub-family on the basis of their similarity score. The analysis concerned all the detectable cavities in NRs, with particular attention paid to the active site pockets. This approach can guide the investigation of NR intrinsic dynamics, the selection of reference structures to be used in drug design and the easy identification of alternative binding sites.

## 1. Introduction

Nuclear receptors (NRs) constitute one of the largest families of eukaryotic transcription factors (TFs) that regulate the expression of genes that control a broad spectrum of physiological functions, including cell development, reproduction, homeostasis, and metabolism [1,2,3]. For their key role, NRs are of great interest in biomedical research and drug discovery, as they are involved in multiple diseases, such as cancer, inflammation, and metabolic diseases [4,5,6]. Indeed, they account for 3% of all human drug targets and are responsible for the therapeutic effect of 16% of all marketed drugs [7].

Based on alignments and phylogenetic tree reconstruction methods, the NR superfamily has been divided into 7 sub-families, numbered from 0 to 6, for a total amount of 48 NRs [8]. With the only exception of the atypical members SHP and DAX, belonging to the NR0 sub-family, all NRs share a common modular organization composed of five domains (A–E) with specific functions. Due to the high mobility, full-length nuclear receptors are difficult to crystallize, and the only accessible domains are the most rigid portions, i.e., the ligand-binding domain (LBD) and the DNA-binding domain (DBD). Of these, the LBD is responsible for the binding of exogenous and endogenous molecules, for receptor homo- and hetero-dimerization and for the interaction with coregulatory proteins; it is characterized by a relevant flexibility and can adopt different conformations according to the bound ligand [9,10].

Flexibility is an inherent feature of proteins, as it is essential for their biological function. It is also synonym of structural adaptability, which reflects the ability of a protein to undergo conformational adjustments to better fit the binding of molecules or as part of its mechanism of action [11]. This is particularly true for NRs, which base their biological function on the ability to stabilize (or displace) a short α-helix segment (H12) in (or from) its active conformation in a ligand-dependent fashion [12]. The flexible nature of H12 has been investigated over the years, leading to the postulation of different models for NR activation. The first “mouse-trap” model [13] hypothesized that, in the absence of a ligand, H12 is in a fixed extended position outside the LBD, referred to as the inactive conformation. As a result of ligand binding, H12 moves towards the LBD, entrapping the ligand and defining the active conformation (Figure S1). Later, Schwabe and co-workers reported the “H12 dynamic stabilization” model, according to which H12 is highly flexible in the apo state and its mobility, as well as the overall LBD mobility, is reduced upon ligand binding [14]. Hence, NRs exist in a dynamic equilibrium between the inactive and the active state and a disorder-to-order transition is induced by the ligand. Depending on the nature of the ligand, H12 spans across distinct conformations; typically, agonist molecules stabilize H12 in the active conformation, promoting the recruitment of co-activators, while antagonists sterically prevent the activation (Figure S1). Moreover, subtle changes in H12, induced by partial agonists, can occur, and affect the recruitment of coactivators and the consequent activation of the transcription machinery [15,16,17,18,19,20]. Indeed, the different NRs can undergo a diverse range of flexibility. Some of them, as ERα, RARα or PR, can explore both agonist and antagonist conformations, while others seem to explore a more reduced conformational space, as LRH-1 and SF-1, having the same H12 conformation when either binding to coactivators or corepressors. The intrinsic dynamics of NRs make their usage in structure-based drug design campaigns quite challenging, especially when it comes to predict flexibility and to select the more representative conformations.

Due to their relevant role in many physiological processes, NRs have been extensively studied for their interaction with ligands and, in the last few years, numerous X-ray structures have been deposited in the Protein Data Bank (PDB), showing a wide range of structural diversity in ligand binding. Taking advantage of this information, a rapid method for the investigation of NRs intrinsic flexibility was applied here and a deep inspection on the adjustments of the LBD was provided.

LBD pockets that belong to each NR sub-family have been calculated with the FLAP*site* algorithm [21] and compared by simply aligning the residues lining them. The comparison retrieved similarity values that provide a general overview on the variability profile of each NR member and facilitate the recognition of the most flexible ones and of putative alternative pockets. Then, to obtain insights on the determinants of the pocket variability, a more detailed level of information was provided for some receptors through clustering analysis. Similar pockets within each sub-family were grouped together, highlighting the key differences within the dataset. This enabled us to map the areas of the LBD of higher plasticity and those more prone to adjustments that can be exploited for the design of new drug candidates. For some receptors, it was also possible to associate specific pocket arrangements with the chemical nature of the bound molecule, as well as the key residues involved in the interaction. Indeed, the peculiar plasticity of LBD binding site in the diverse NRs could be exploited for the design of specific ligands with less adverse effects and less off-targets within the same superfamily [2]. To this aim, we also report a cluster analysis performed on each sub-family for the identification of medoids that represent the most populated conformations assumed by each NR in the Protein Data Bank. These structures could be used for any structure-based drug design campaigns for the identification/development of new ligands, but also for running virtual screening or ensemble docking simulations, aiming to take into account NR flexibility. In addition, a detailed picture of the current structural knowledge on NRs is provided.

With respect to other methodologies that rely on graph theory (clique detection algorithms, etc.) or more complex pocket description, providing diverse and additional information (we refer to other manuscripts for a proper description of the available tools that we do not want to compete with [22,23,24,25,26,27,28,29,30]), this alignment-based method is extremely simple and fast, and can be applied because no variation in the protein sequence is experienced in each sub-family.

## 2. Results

The procedure applied here consists, mainly, of the following three parts: (i) selection of NR structures from PDB, (ii) pocket detection, (iii) pocket comparison and data elaboration. All the NR LBD structures present in the PDB were retrieved and retained if they had a resolution of ≤2.5 Å and if they contained a ligand (see Section 4 for further details and Table 1 for the complete list). A few receptors, either lacking structural information or with no sufficient data, were excluded from the analysis. The latter largely belong to the NR2 family and partly to the NR1 and are referred to as orphan receptors because no endogenous ligand has been identified for them.

The selected structures were submitted to the FLAP*site* calculation [21] for the identification of accessible cavities within the individual proteins. With the purpose of providing an estimation of the local structural variability regarding the LBD, only the binding sites hosting putative ligands, hereafter liganded pockets, i.e., active sites and allosteric sites, were considered for the following steps.

The FLAP*site* algorithm returns the accessible pockets as PDB files and the corresponding indices of residues lining them. Only this very simple final information, returning the entity of lining residues, along with their sequence number (residue index), has been used. Thus, by verifying that the numeration of the sequence residues is maintained for the same receptor, cavities comparison is purely performed on the basis of the residue index in the sequence. Then, within each NR sub-family, all possible pairs of pockets were compared, checking the number of shared residues over the total number of residues that make up the pockets (see Equation (1) in Section 4). The number of conserved residues for a pair of pockets was defined as a ”similarity value” (or “*S value*”); an *S value* of 0 indicates no correspondence in the residue indices and refers to completely different cavities that are likely to be located in distinct regions of the protein. An *S value* equal to 100 means the pockets are identical, while decreasing *S values* indicate a more or less important difference in a pair of pockets, attributable to a different pocket shape (larger or smaller pockets will present different lining residues in terms of nature and quantity) or to mutations occurring at the binding site. Thus, sub-families for which *S values* span across a wide range present a relevant degree of variability at the binding site, while others with more consistent values have geometrically more conserved pockets.

### 2.1. Intrafamily Variability

The degree of binding site variability was determined for each sub-family with at least three ligand-bound structures that met the selection criteria. Sub-families that presented a lower number of structures were not included in the analysis. To provide an estimation of the overall variability for each of the five families, the *S values* of the corresponding NRs were collected and elaborated in the boxplot form, where the box length reflects the similarity of a receptor’s binding site within the different PDB coordinates, i.e., the longer the box, the higher the variability. Here, the boxplots for classes NR1, NR2, NR3, NR4 and NR5 are reported. No structural data were available for family NR6, which, therefore, is not included in the analysis.

#### 2.1.1. NR1. Thyroid Hormone Receptor-like Family

**Figure 1:**
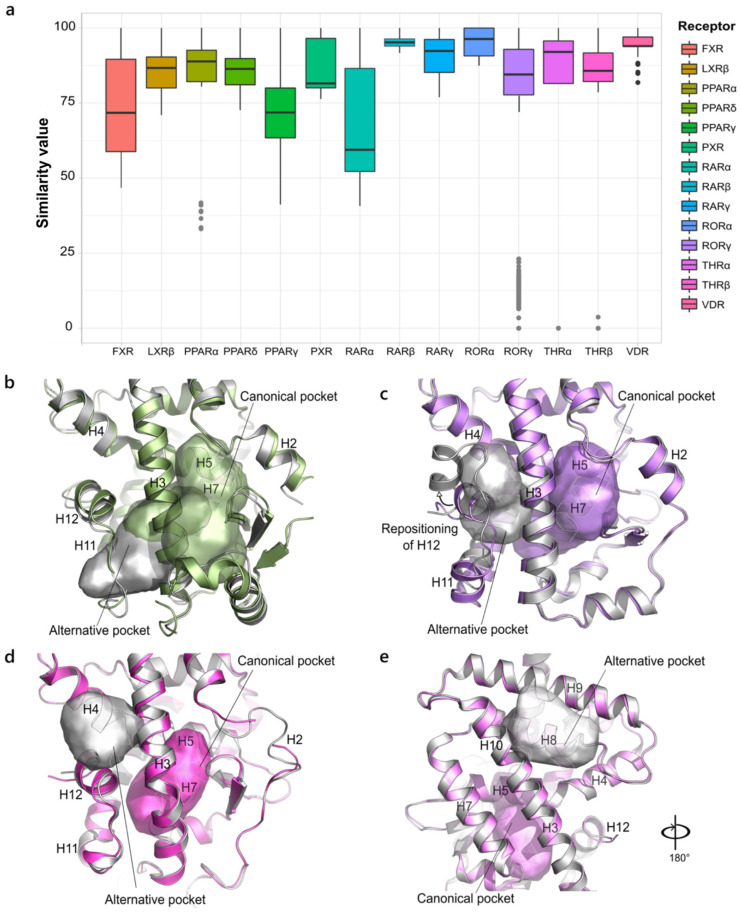
Variability in NR1 family. (**a**). Boxplot representing the structural variability in the LBD binding site in the NR1 family. The outlier points corresponding to non-canonical binding pockets are highlighted in grey. (**b**). Superposition of the canonical active pocket (green, PDB ID 2P54) and the outlier one (grey, PDB ID 5HYK) in PPARα. The two pockets are intersected, as they share some of the residues in helices H3, H5 and H7. (**c**) Superposition of the canonical active pocket (lilac, PDB ID 5NIB) and the outlier one (grey, PDB ID 5C4S) in RORγ. The newly induced orientation of H12 is indicated by an arrow. (**d**) Superposition of the canonical active pocket (magenta, PDB ID 2J4A) and the outlier one (grey, PDB ID 2PIN) in THRβ. (**e**) Superposition of the canonical active pocket (violet, PDB ID 2H79) and the outlier one (grey, PDB ID 4NLW) in THRα.

At a first glance, FXR, PPARα, PPARγ and RARα exhibit the largest range of *S values*, and outlier points are visible for PPARα, RORγ, THRα and THRβ (Figure 1a). The very low score of outlier points implies that none or very few residues are shared between the mentioned pockets and those included within the box plots, indicating a distinct targeted area.

In PPARα, the outlier points correspond to a pocket close to the canonical one but are confined within H3 and H11, in which a smaller and more rigid ligand can be accommodated [31] (Figure 1b). The new pocket exhibits a similarity score in the range of 35–50% with the rest of the dataset, as it shares residues in H3, H5, H12 and the bottom part of H7. In RORγ, the outlier points correspond to an alternative pocket that shifted toward H12 and is distal with respect to the orthosteric cavity (Figure 1c), generally occupied by allosteric inverse agonists that induce a new orientation of H12 that prevents co-regulator recruitment [32]. In THRβ, the outlier cavity refers to the AF-2 pocket, delimited by H3, H4 and H12, typically bound by the co-regulator peptide. An antagonist molecule is found in this pocket, acting as a direct inhibitor of the co-activator recruitment, without interfering with H12 conformation [33] (Figure 1d). In THRα, the outlier points indicate an unprecedented binding site located between H8, H9 and H10. The natural ligands, T_3_ and T_4_, were found to bind also in this pocket that could be exploited as a new target site (Figure 1e) [34].

Despite the very low number of structures available in the PDB (only four), RARα is among the receptors with the largest similarity range, being co-crystallized with agonists, antagonists [35] and inverse agonists [36] that lead to diverse conformations of the ligand binding pocket (LBP). In contrast, both subtypes RARβ and RARγ show a conservation range within 75%, as their structures are all in agonist-like conformations and are bound by similar ligands. Within the same conservation threshold, there is RORα, whose very poor dataset (three structures) might contribute to the small value range. On the other hand, the larger dataset of VDR, only composed of agonist conformations, still reflects a high conservation of the LBP.

Of the two isoforms of the liver X receptor (LXR), only LXRβ is counted because of the insufficient number of LXRα structures suitable for the analysis. All the available crystal structures of LXRα lack a substantial portion of H1, which is part of the binding pocket, with the only exception of PDB ID 3IPU, where the bigger size of the ligand enables direct interactions with the helix so as to stabilize it and enable the crystallization [37]. On the other hand, the seven X-ray structures of the β isoform show a range value within 70%, due to slight differences in the dimension of co-crystallized ligands that are reflected in the slight differences of the pocket size. As well as the previously mentioned receptors, the *S value* of PXR is always higher than 70%. Although the ligands are quite dissimilar in terms of structure and orientation within the binding site, the PXR pocket is quite conserved in the agonist conformation and only registers the mismatch of very few residues.

The isoform δ and γ of PPAR are characterized by a large binding site that often hosts multiple ligands and allows branched and different molecule sizes to bind. The number of PPARγ structures examined here is notably reduced compared to the starting number, as it has been removed from the large number of X-rays that lack the H2′-H3 loop. The latter is typically very flexible and not always observed in the electron density maps but constitutes an integral part of the binding pocket. The H2′-H3 loop is indeed the main factor responsible for pocket variability in both PPARδ and PPARγ, as its different conformations across the X-rays results in a different composition of the residues that encompass the binding site. In PPARδ, for instance, the main difference is attributed to a group of pockets where the H2′-H3 loop approaches H3, thus allowing the extension of the binding pocket around it [38]. On the other hand, in PPARγ, the greater dissimilarity is additionally due to different size molecules and to the presence of partial agonists that typically bind to only half of the binding site.

#### 2.1.2. NR3. Estrogen Receptor-like Family

**Figure 2:**
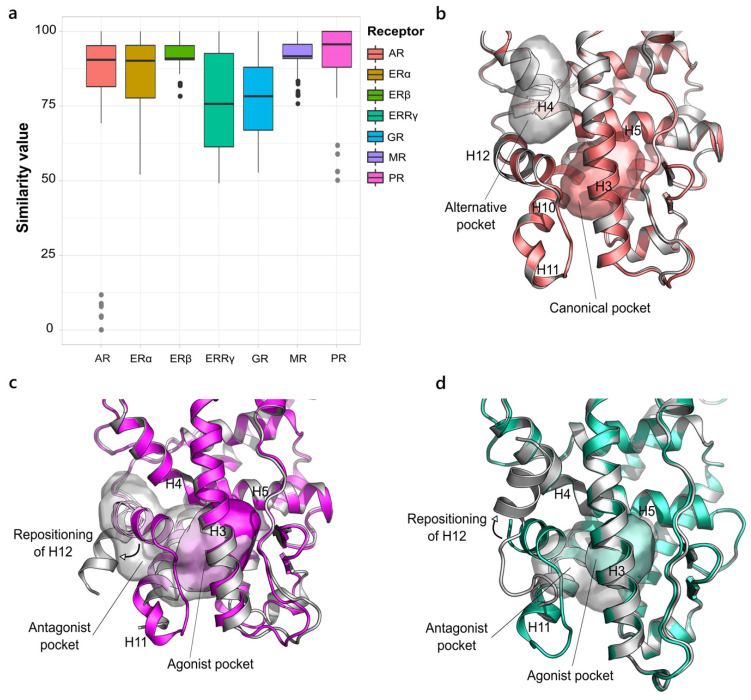
Variability in NR3 family. (**a**) Boxplot representing the structural variability in the LBD binding site in the NR3 family. The outlier points corresponding to non-canonical binding pockets are highlighted in grey. (**b**) Superposition of the canonical active pocket (salmon) and the outlier one (grey) in AR (PDB ID 2PIP). (**c**,**d**) Superposition of the agonist pocket of PR (magenta, PDB ID 1A28) and ERRγ (green, PDB ID 2P7G) and the antagonist pocket in grey, (PDB IDs 2OVH and 2GPU, respectively). The two pockets are intersected as they share residues in H3, H5 and H10. The rearrangement of H12 from the agonist to the antagonist conformation is highlighted.

Overall, the NR3 family displays a reduced variability, within 40% of similarity, compared to family 1 (Figure 2a). Two receptors, AR and PR, are marked by more evident outlier points. Among all the agonist-like X-ray structures of AR, a few are characterized by the presence of molecules in sites different from the canonical ligand-binding site. In particular, the outlier points in the boxplot correspond to the cavity where co-activators bind, namely the AF-2 pocket, located far from the standard ligand binding pocket (Figure 2b). In some X-ray structures, the same small molecules that bind to the AF2-pocket have also been found in a novel additional pocket between H1, H4 and H9, called binding function 3 (BF3) [39], which is thought to allosterically influence co-regulator recruitment. We were not able to detect this new site with our pocket search algorithm, likely due to the fully solvent exposed location that affects its identification, but we mention it for completeness. The case of PR stands in contrast, where one structure is bound by an antagonist molecule and is, therefore, arranged in an “open state” in which the size of the canonical pocket is doubled (Figure 2c). As the only deposition in the antagonist conformation, the related similarity scores are assessed as outliers. Together with ERα and GR, which will be discussed in detail, ERRγ presents one of the widest *S value* ranges, as it is crystallized with both agonist and antagonist ligands. Again, the comparison of the pockets in both conformations clearly shows the doubled size of the open state pocket with respect to the closed one (Figure 2d). ERβ and MR show the most conserved pockets. The few outlier points in ERβ describe a group of structures in which H12 is arranged in a conformation that looks similar to an intermediate state between the open and the classical ER’s closed one, potentially induced by partial agonists [40]. As a result, the pocket size is overall unaffected and still maintains a high degree of similarity with the canonical one. In MR, the outlier points refer to a slight enlargement of the binding site, which contains a couple more residues, in response to the binding of bulkier ligands.

#### 2.1.3. NR2, NR4 and NR5. Retinoid X Receptor-like, Nerve Growth Factor IB-like and Steroidogenic Factor-like Families

**Figure 3:**
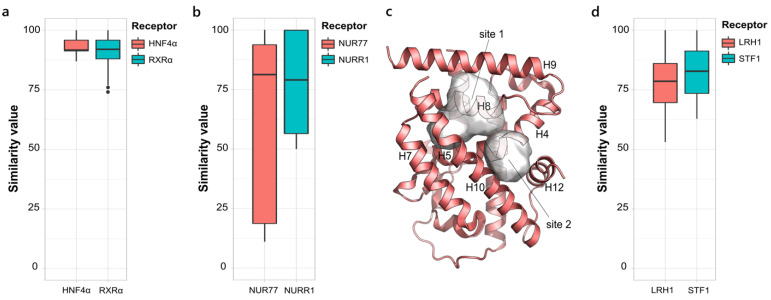
Boxplot representing the structural variability in the LBD binding site in the NR2 family (**a**), the NR4 family (**b**) and the NR5 family (**d**). (**c**) The two alternative pockets site1 and site2 (PDB IDs 4R38 and 3V3Q, respectively) in NGFI-B, belonging to the NR4 family.

Despite being the second most populated NR family, the retinoid X receptor-like family (NR2) only accounts for a few X-ray structures and only two of them fulfilled the desired criteria to be analyzed. The distribution of the *S values* for HNF4α and RXRα indicates strongly conserved binding sites for both the receptors (Figure 3a). However, the very reduced size of the HNF4α dataset and the chemical similarity of the co-crystallized ligands likely affected the analysis outcome. Even if molecules binding to RXRα show greater heterogeneity, good similarity among the binding pockets is still registered. The typical L-shaped cavity can, indeed, accommodate diverse molecules that span from agonists to partial agonists [41] to heterodimer-specific ligands [42], while preserving the main structural features for ligand recognition by RXRα.

The nerve growth factor IB-like family (NR4) is a family of ligand-independent nuclear receptors classified as immediate early response genes, induced by multiple stressors [43]. No cavity is found within the canonical ligand binding site, as it is filled with bulky residues, as well as no classical binding site for coactivators can be observed. However, the binding of molecules in alternative pockets has been reported for NGFI-B and Nurr1 and their comparison is shown in Figure 3b. In particular, two adjacent sites, site 1 (H5, H8, H9, H10) and site 2 (H4, H11, H12), are identified in NGFI-B, with a very low similarity score (~15%) between each other (Figure 3c). Molecules binding to the two pockets directly affect the association of NGFI-B with its interacting partners, either enhancing [44] or blocking it [45], and resulting in different biological outcomes and therapeutic applications. Three crystallographic structures comprising Nurr1 and activator molecules, found to be considerable for the development of synthetic ligands to treat Parkinson’s disease, are available. These molecules all bind to the non-canonical binding site located between H4, H11 and H12 through the establishment of a covalent bond with Cys566. Due to the largely different size of the binding ligands, one pocket has a double volume compared to the others, thus extending the conservation range to 50%.

The NR5 family comprises the steroidogenic factor-1 (SF-1) and the liver receptor homologue-1 (LRH-1), two constitutively active receptors that, in contrast to canonical NRs, are arranged in an active conformation, even in the absence of a ligand and whose activity can depend either on the ligand, on post-translational modifications, or on the co-regulators expression levels in a specific tissue [46]. Both the receptors have a very large and hydrophobic ligand binding cavity that is often occupied by bacterial phospholipids from crystallization. The corresponding boxplot is shown in Figure 3d. The four crystal structures available for SF-1 are all bound by PLs that expose the negatively charged head to the solvent, and only very slight differences in the binding pocket are registered. The same typical binding mode of PLs is adopted in LRH-1, which has also been crystallized with synthetic small agonist compounds. Despite the markedly different size of PLs and synthetic molecules, no large differences in the composition of the pocket are observed, while the volume significantly changes. Indeed, the binding of small molecules results in a narrowed cavity, due to the shift of H6 and H3 towards the inside of the cavity.

### 2.2. Intra-Sub-Family Variability

To look more in detail at the variability in each sub-family, all the retrieved pockets that belong to each single receptor were clustered. Because of the used hierarchical clustering algorithm (see Section 4), the similarity matrices, containing the *S values* of all the pairs of binding sites, were transformed into dissimilarity measures. In such a way, the meaning of the score values is inverted, i.e., 0 is a synonym of maximum identity between the pockets and 100 indicates the largest disparity. Consistent with the information retrieved from the boxplot, the higher the dissimilarity is between the clusters, the higher the variability within the receptor’s binding site. This diversity is directly correlated to the diversity of the co-crystallized ligands and the related induced conformational changes in the binding site.

A selection of the most relevant examples, according to the pocket variability and to the number of available structures, is reported hereafter. The data for the other sub-families are reported in the Supplementary Information (Figures S2–S4).

#### 2.2.1. Androgen Receptor (NR3 Family)

**Figure 4:**
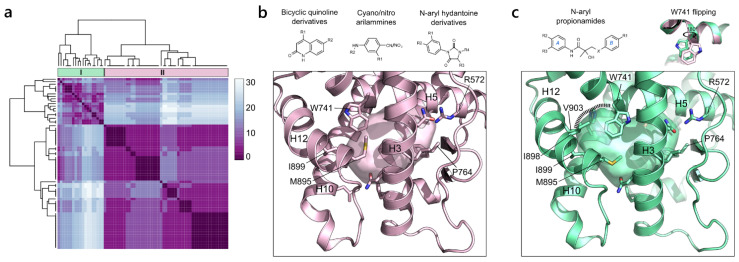
AR variability. (**a**) Heatmap of the hierarchical clustering of AR. (**b**) On the bottom, the representation of the pocket from cluster II (PDB ID 1T63) is shown and on top, the general chemical structures of the binding compounds are shown. (**c**) On the bottom, the representation of the pocket in cluster I (PDB ID 3B67) is shown. On the top, the chemical structure of the N-aryl propionamide compounds and the flipping of W741 responsible for the extension of the binding site in the WT form of AR are shown.

AR plays a critical role in metabolism and reproduction and is the main target for the development of prostate cancer treatments [47]. In the last few years, numerous selective androgen receptors modulators (SARMs) have been developed as a class of therapeutic agents that can exert both agonist and antagonist effects on AR in different tissues [48]. To, date all the crystallographic structures of AR present the same general agonist-like conformation, with H12 in the closed state. As a result, the PDB is rich in agonist structures of AR complexed with diverse SARMs in the wild-type form, and of specific mutated forms that allow the crystallization of antagonist molecules in the active agonist-like conformation. In particular, the T877A and W741L mutants, derived from the prolonged treatment with potent anti-androgenic drugs for prostate cancer (hydroxyflutamide and R-bicalutamide, respectively), are responsible for converting antagonists to agonists [49]. To retrieve the major differences among the canonical ligand-binding sites of AR coordinates, the outlier pockets that referred to the alternative sites previously discussed were removed from the hierarchical clustering analysis. The similarity of the canonical pockets ranges from 70% to 100%, indicating quite strong similarity. Despite this, it is possible to identify two clusters of pockets as a result of the ligand-induced fit and to distinguish among the different SARMs chemical classes (Figure 4a). On the one hand, the larger cluster (II) refers to the canonical binding pocket, bound both by the endogenous androgens (testosterone and dihydrotestosterone) and by non-steroidal SARMs of comparable size (quinolone derivatives, cyano(nitro) arylamines, N-aryl hydantoine derivatives). This pocket extends from Arg572 on H5 and Phe764 on the β-sheet to Met895 and Ile899 on H12 (Figure 4b). On the other hand, the smaller cluster (I) refers to larger pockets and contains both the AR mutants and the WT AR in complex with N-aryl propionamides, the bulkiest compounds among the SARMs classes, fully occupying the available room in the ligand binding pocket. The expansion of the pocket is ruled by Trp741, which, except for the W741L mutants, undergoes a flipping of the side chain to accommodate the differently modulated B-ring of the aryl-propionamides. The resulting expanded pocket includes additional residues on H12, namely Ile898 and Val903, and His874 on H10 (Figure 4c).

#### 2.2.2. Glucocorticoid Receptor (NR3 Family)

**Figure 5:**
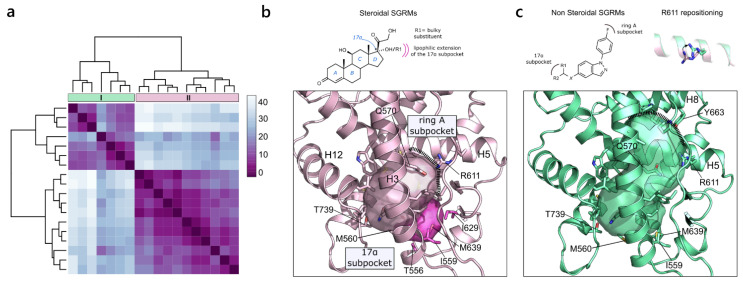
GR variability. (**a**) Heatmap of the hierarchical clustering of GR. (**b**) On the bottom, the representation of the pocket from cluster II (PDB ID 4UDC) is shown. A small extension of the canonical pocket is highlighted in magenta (PDB ID 4UDD). On top, the chemical structure of the endogenous ligand, cortisol, is shown, indicating the position for the modulation of steroidal SGRMs. (**c**) Representation of the extended pocket from cluster I (PDB ID 4CSJ). On the top, the general chemical structure of non-steroidal SGRMs and the flipping of R611, enabling the extension of the binding site, are shown.

The hierarchical clustering of the active pockets of the glucocorticoid receptor (GR) clearly shows the existence of two clusters (Figure 5a). Similar to AR, the binding sites are partitioned accordingly to the chemical nature of the ligands, ranging from endogenous ligands to steroidal and non-steroidal selective glucocorticoid receptor modulators (SGRMs). The most populated cluster (cluster II) corresponds to the canonical pocket bound by steroidal compounds, which is bordered on the top by the two gatekeeper residues, Arg611 and Gln570, and on the front bottom by Thr739 and Met560. These two delimiting portions are commonly referred to as the A-ring and 17α regions, respectively, from the chemical structure of the endogenous ligand cortisol (Figure 5b). Within the same cluster, we find pockets that are slightly enlarged at the ligand 17α region. This small sub-pocket, confined between H3 and H7, is mainly hydrophobic and includes the addition of Ile559 and Thr556 on H3, Met639 and Cys643 on H7 and Ile629 on the β-sheet. Cluster I includes X-ray structures of GR in complex with the larger non-steroidal SGRMs that occupy new regions of the LBP by enlarging the pocket dimension. The extended cavity reaches, on the bottom, the already mentioned 17α sub-pocket and, on the top, opens to a sub-pocket in the A-ring region, after the repositioning of Arg611 (Figure 5c).

#### 2.2.3. Estrogen Receptor α (NR3 Family)

**Figure 6:**
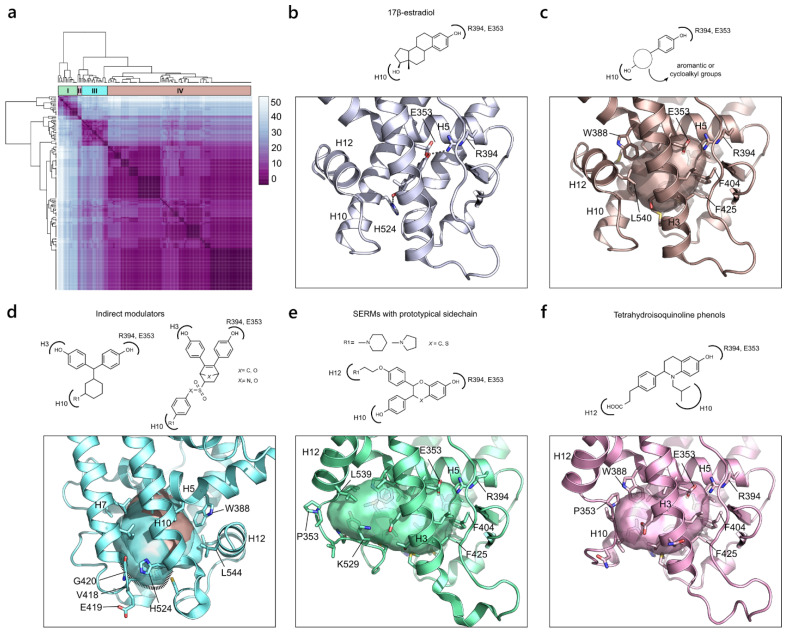
ERα variability. (**a**) Heatmap of the hierarchical clustering of ERα (PDB ID 3UUD). (**b**) Illustration of the binding mode of the endogenous ligand, 17β-estradiol, in ERα. The main polar interactions are shown with dashed lines. (**c**) Representation of the canonical pocket resulting from cluster IV (PDB ID 1L2I). On the top, a generic formula of the binding compounds is shown. (**d**) Depiction of the pocket described by cluster III (PDB ID 5DUH). The receptor is rotated by 180° to highlight the orientation of His524, pushed out from the cavity because of the ligand bulky substituent pointing at it. On top, the chemical structures of the indirect modulators are shown. (**e**) Representation of the antagonist pocket retrieved from cluster I (PDB ID 2AYR). On top, the chemical structure of the common antagonist molecules that possess a prototypical side chain is shown. (**f**) Representation of a different antagonist pocket induced by a specific class of SERD molecules (tetrahydroisoquinoline phenols) obtained from cluster II (PDB ID 5FQP).

The estrogen receptor α (ERα) accounts for the highest number of X-ray structures among the whole NR superfamily, as it has been extensively studied for its role in ovarian and breast cancer [50]. The clustering of the final 148 pockets returned 4 main groups (Figure 6a). The largest cluster (cluster IV) accounts for most of the canonical pockets with H12 in agonist conformation. This pocket is mainly lipophilic, with a few chances for polar interactions that can be exemplified by the binding of the endogenous ligand, 17β-estradiol (Figure 6b). The most conserved interaction, across all the chemical classes of compounds binding to ERα, is guaranteed by a small hydrophilic area defined by Arg394 on H5 and Glu353 on H3. Another interaction, common in many ERα agonists, is made with His524 on H10. Overall, the pocket is bordered on one side by Phe404 on the β-sheet and Phe425 on H7, and on the other side by Trp383 on H5 and Leu540 on H12 (Figure 6c). Within the same cluster, small modifications in the chemical structure of the agonist compounds lead to small adaptations of the pocket, particularly in the lipophilic region at the β-sheet extremity, which can be filled by bulky substituents, and at the H12 extremity. Overall, these minimal modifications do not affect the architecture of the pocket, preserving a high similarity score. While maintaining an agonist conformation, cluster III differs from the canonical pocket, as it includes a group of molecules referred to as “indirect modulators”, a class of selective estrogen receptor modulators (SERMs) that antagonize ERα through an alternative mechanism [51]. Chemically, these compounds contain a bulky substituent, which points at H10, perturbing the secondary structural elements and provoking a shift of H11 that, indirectly, regulates the dynamics of H12. The resulting pocket is enlarged and counts the additional residues, Val418, Glu419 and Gly420, where the bulky groups point at and interact with (Figure 6d). Finally, the last two clusters (I and II) refer to the classical antagonist pocket where the H12 switch impedes the co-activator recruitment. Despite the fact that both clusters describe the same conformational rearrangement, differences are observed. Cluster I comprises molecules with diverse antagonist profiles ranging from SERMs to selective estrogen receptor α modulators (SERAMs) and selective estrogen receptor down-regulators (SERDs). They all possess a prototypical sidechain, with a pyrrolidine or pyperidine function, which protrudes against H12, inducing the enlargement of the pocket. The resulting pocket accounts for the addition of all the residues on the loop connecting H10 and H12 (from Lys529 to Pro535) and extends until Leu539 on H12 (Figure 6e). In contrast, cluster II represents a slightly smaller antagonist pocket as it contains SERD molecules with a shorter prototypical chain that provokes an introflection of the loop towards the binding site. This means that not all the residues on the loop contribute to the definition of the pocket, which, in turn, extend until Pro535 without reaching H12 (Figure 6f).

#### 2.2.4. Retinoic Acid-Related Orphan Receptor γ (NR1 Family)

**Figure 7:**
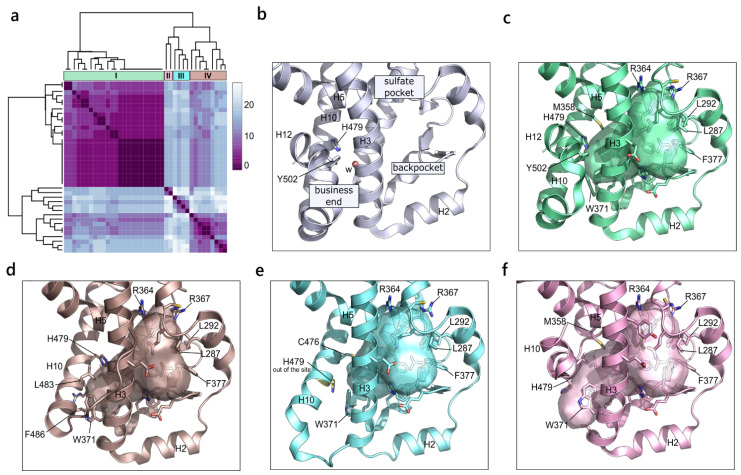
(**a**). RORγ variability. Heatmap of the hierarchical clustering of RORγ (PDB ID 5NI5). (**b**) Illustration of the RORγ binding site. The main areas of the cavity are labelled. (**c**) Representation of the pocket described by cluster I as a result of the binding of agonist molecules or inverse agonist acting via the “water trap” mechanism (PDB ID 3KYT). (**d**) Pocket resulted from cluster IV and induced by inverse agonist acting via the “push and pull” mechanism (PDB ID 4WQP). (**e**) Representation of the pocket of cluster III. H479, pushed out of the binding site, is highlighted in yellow (PDB ID 5 × 8Q). (**f**) Depiction of the pocket described by cluster II (PDB ID 6Q2W).

To obtain insights on the different configurations of the canonical pocket of the retinoic acid-related orphan receptor γ (RORγ), the alternative cavities were discarded from the following analysis and the similarity window was narrowed to the 70–100% conservation range. The related heatmap, with the four identified clusters, is shown in Figure 7a. The first cluster represents pockets that are typically bound by agonist molecules with H12 in the active conformation, stabilized by the hydrogen bond between His479 and Tyr502. This cluster also includes a class of inverse agonists that bind to the agonist conformation and that are thought to act via a water-trapping mechanism [52].

A water molecule is indeed found at the so-called “business end” region of the active site in a partly hydrophobic environment, and its release into the solvent, with the following gain of free energy, is supposed to destabilize H12 (Figure 7b). Overall, the pocket described by cluster I, corresponding to the agonist conformation, extends from the β-sheet to the front end where it is bordered, on the four sides, by Tyr502 on H12, His479 on H10, Trp371 and Leu324 on H3 and Met358 on H5 (Figure 7c). Cluster IV refers to constructs without H12 as a result of the co-crystallization with inverse agonists. In fact, such ligands can destabilize H12 by H-bonding to His479, moving it from its favorable position for the interaction with Tyr502. As a result, in the absence of H12, H10 bends towards the binding site, now bordered by Trp371 and Leu324 on H3, and by His479, Leu483 and Phe486 on H10 (Figure 7d). A further shift of H10 towards H3 appears in cluster III, where the inverse agonists sterically clash into His479, pushing it out of the pocket (Figure 7e). Finally, a closure of the binding site is observed in cluster II, where ligands induce a twist in H10, closing the cavity. This orientation greatly disturbs the organization of H11 and H12, which cannot be resolved in the crystal structure (Figure 7f).

#### 2.2.5. Farnesoid X Receptor (NR1 Family)

**Figure 8:**
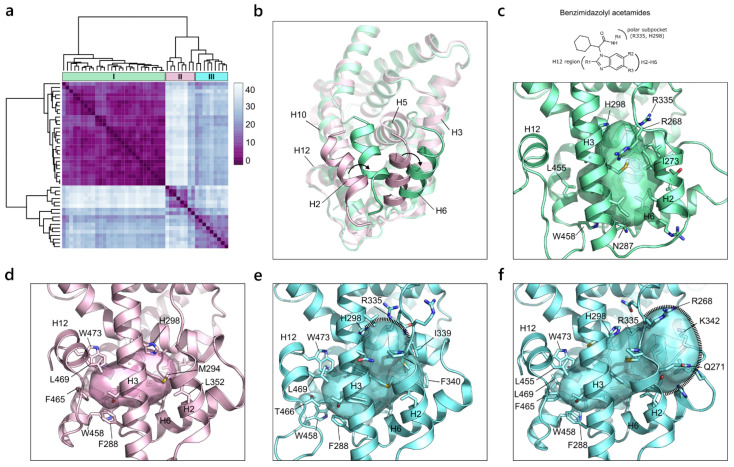
(**a**) Heatmap of the hierarchical clustering of FXR. (**b**) Superposition of FXR structures containing H2 and H6 in the canonical (pink, PDB ID 5ICK) and non-canonical (green, PDB ID 5Q1H) conformations. (**c**) Representation of the pocket defined by cluster I (PDB ID 5Q1H) as a result of the binding of compounds that contain a benzimidazolyl acetamide scaffold (reported on the top). (**d**) Depiction of the pocket illustrated by cluster II (PDB ID 5ICK). (**e**,**f**) Representation of the two expanded pockets described by cluster III (PDB IDs 5Q0W and 6A5Y, respectively).

From the hierarchical clustering of the farnesoid X receptor (FXR), four main types of pockets were identified. The heatmap representation highlighted the presence of a large and compact cluster, containing more than the half of the analyzed binding sites, and other smaller clusters (Figure 8a). The first group of pockets is derived from a set of crystallographic structures characterized by the shift of helices 2 and 6 towards the left, with respect to the canonical position (Figure 8b). This structural rearrangement occurs as an induced fit mechanism to prevent the steric clash of bulkier ligands with H6 [53]. Indeed, cluster I contains X-ray structures whose co-crystallized ligands show a common scaffold and a similar bulky substituent. Additionally, they all have a polar substituent that points to a small hydrophilic region delimited by His298 and Arg335, which, in turn, define the upper boundary of the first group of pockets (Figure 8c). The other clusters in the heatmap contain FXR structures with H2 and H6 that occupy the canonical position, and co-crystallize with ligands of different size that induce different alterations in the binding site volume. Cluster II represents the smallest pocket size, as all the co-crystallized molecules are small and mainly occupy the area of the binding site towards H12, without extending beyond H5. This results in a small cavity bordered on one side by Trp473 and Leu469 on H12, and on the other side by Met249 on H3, Leu352 on H6 and His298 (Figure 8d). By increasing the molecule size, a consequent enlargement of the pocket is observed in cluster III, which contains two different representations of the ligand binding site. On the one hand, a polar substituent pointing at H3 expands the pocket to the small hydrophilic area bordered by Arg335. On the other hand, substituents pointing between H2 and H6 further extend the pocket in that direction, counting the addition of Arg268, Lys342 and Gln271 (Figure 8e).

### 2.3. Identification of Representative Structures

The cluster analysis enabled us to identify groups of pockets with slight or major differences that indicate different depictions of the same binding site. These multiple representations could be used in SBDD campaigns to achieve a more complete characterization of a specific receptor. To obtain a pool of representative structures for each NR, we computed the medoids of each cluster for each receptor, as listed in Table 2.

## 3. Discussion

Many NRs have been crystallized and their structure deposited in the PDB in the last two decades. Among these, the most studied NRs have been those involved in relevant diseases, such as ERα and AR, as targets for breast and prostate anticancer therapeutics [54,55,56,57], respectively, but also, in the same NR3 family, GR, which is a relevant target for inflammatory diseases [58]. In NR1, the most studied receptors include FXR, RORγ, PPARs and VDR, and RXRα in NR2 [59,60,61,62,63,64,65,66]. In a few of these cases, higher binding site plasticity has been observed and was first attributed to the highest number of available crystal structures. However, discrete variability has been also observed for the less studied receptors, such as ERRγ or RARα, for which the presence of co-crystallized ligands with different sizes and different binding modes allowed higher pocket flexibility. Thus, there is apparently no direct correlation between the LBD flexibility and the number of available X-ray structures. On the contrary, intrinsic dynamics seem to be a specific feature of each single receptor, and, in fact, not all of them are able to undergo the agonist-antagonist-like conformational switch. For instance, AR, which has been widely and extensively studied, has not yet been crystallized in the antagonist conformation, possibly because of the higher flexibility or instability of this form [67].

In a recent study performed on many ERα structures, Schneider et al. confirmed that the most relevant dynamic adjustment is related to H12 reorientation [68], while much smaller fluctuations have been observed for the rest of the protein. ERα complexes with agonists and antagonists, characterized by different H12 orientations, were, thus, easily separated by clustering and, in both cases, limited adjustments were observed in loops. MD simulations performed on three complexes highlighted the same regions as the more flexible residues, thus supporting the X-ray findings. In particular, the most flexible residues have been identified as Met343, Met421, His524 and Met528, and the inclusion of their side-chain flexibility produced better results when screening both agonist and antagonist molecules. Interestingly, these residues appear to be the most flexible also in the structures we selected as the most representative structures for ERα (including two agonist-like and two antagonist-like conformations, Figure S5). Particularly crucial seems to be the rearrangement of His524 that allows for the accommodation of bulkier ligands, regardless of their agonist and antagonist nature. It suggests that the similarity value-based clustering that we adopted could represent a valuable tool for selecting effective structures to be used in virtual screening and docking campaigns, avoiding the generation and the use of in silico conformations, at least for the most populated NRs. Indeed, when performing virtual screening simulations on a set of X-ray and MD structures, the authors found that crystal structures performed better than computationally generated ones, confirming previous findings obtained for AR [69].

The analyses carried out here allow the rapid identification of the most flexible residues and, in particular, the fast and easy identification of reference structures to be used in virtual screening campaigns for any other NRs sufficiently represented, without the need of running more extensive and demanding calculations [70]. The presented methodology, in fact, only relies on the comparison of pockets based on the ID of the lining residues without paying attention to their chemical characteristics and making the calculation quite fast. To check whether important information was neglected, we compared our approach with the more elaborated and fine technique based on GRID molecular interaction fields (MIFs). In particular, we exploited the BioGPS suite, which allows us to characterize and compare datasets of pockets through MIF calculations [21], taking as an example the case of GR pockets. After calculating the MIFs for the pocket set, we performed a principal component analysis (PCA), obtaining the score plot reported in Figure S6. In line with the previous cluster analysis (Figure 5a), two main groups of pockets were detected, including cluster II that corresponded to the canonical pocket and cluster I that corresponded to the enlarged ones. These results indicate that the two approaches match in detecting clusters of similar pockets, meaning that, despite ignoring the chemical properties of the pocket, no loss of information is registered. More importantly, the computational time is drastically reduced.

## 4. Materials and Methods

### 4.1. Dataset Preparation

The entire study was conducted on the dataset derived from the collection of the human nuclear receptors’ X-ray depositions in the Protein Data Bank (PDB) until 7 June 2019. Only PDB structures that corresponded to the ligand binding domain (LBD) and with a resolution lower than 2.5 Å were used and a minimum of two deposited coordinates was required for a receptor to be considered for the analysis. Allowed exceptions to the resolution restriction were those NR members that contained only crystal structures above the fixed limit but were not higher than 3.5 Å. The PDB entries following these criteria were then collected for processing with the *Fixpdb* tool, implemented in FLAP [71], which enables protein pre-treatment and refinement (e.g., ions and water molecule removal). Subsequently, the size of the dataset was further reduced by removing all the inactive conformations of helix 12 and the 3D structures that lacked the most relevant parts in defining the canonical binding site (integrity criterion). In this regard, particular attention was paid to helix 2, helix 11, the bottom part of helix 3 and 10, and helix 12. For this step and the following steps, multiple chains in the same PDB model were treated as different entities.

### 4.2. Pocket Detection

The FLAP*site* algorithm, developed by Molecular Discovery Ltd., was used for the identification of cavities in 3D protein structures [21]. By embedding the protein structure into a 3D grid with a spatial resolution of 1.0 Å, the algorithm identifies pocket points using the GRID probe H, accounting for the pocket shape. For each point, a buriedness index is calculated. Points with a buriedness index lower than a specific threshold are discarded. Two morphological operations (erosion and dilation) are applied to the remaining points for removing small anomalies and connecting areas. The hydrophobic probe DRY is used to prioritize hydrophobic cavities usually targeted by drugs.

### 4.3. Pocket Comparison

For each of the dataset entries, the pocket finding algorithm FLAP*site* [21,71] was applied. A pocket is defined by the lining residues and each residue has a unique index corresponding to the sequence number in the PDB entry. After cavity detection, pairs of pockets were compared to each other in an all-against-all approach within the same receptor subclass, on the basis of the residue index. Crystal structures that differed in the sequence numbering within the same receptor’s depositions were, therefore, re-numbered to have the index correspondence. Pocket comparison was performed only for the liganded sites and, as a result, a symmetrical square matrix S was obtained for all the nuclear receptors. The value associated to each pair of pockets was defined using the Jaccard index in the percentage form, here named “similarity value” or “*S value*”; for pocket *A* and *B*, the *S value* is expressed as the ratio between the number of common residues (*A*∩*B*) and the total number (*A*∪*B*).

(1) $$S\;value_{AB}=s_{AB}=\left(\frac{N{}^{\circ}\;residues_{A\cap B}}{N{}^{\circ}\;residues_{A\cup B}}\right)\times 100\;0\;<\;S\;value\;<\;100$$

**Figure 9:**
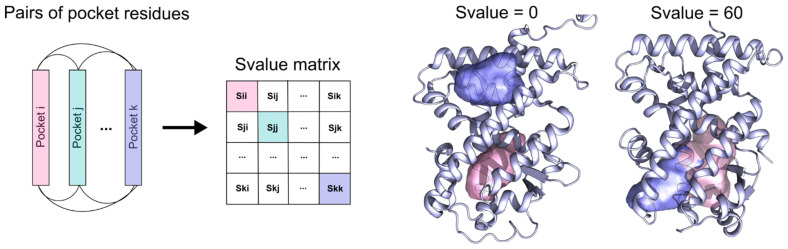
Description of the method. Pairs of pocket residues from the different PDB structures of the same receptor are compared based on the residue index (sequence numbering). The resulting similarity matrix contains all the similarity values (*S values*) of each pair of pockets. An *S value* of 0 indicates pockets that are located far within the protein and have no residue in common, while an *S value* of 60 indicates overlapped pockets that share residues.

The similarity value is defined in the range between 0 and 100 and indicates the degree of similarity between two pockets. A value equal to 0 indicates completely different pockets (no residue correspondence) and 100 indicates strongly conserved pockets (Figure 9).

### 4.4. Data Elaboration

The matrices were finally elaborated with statistical graphic representations and clustering methods using RStudio (version 1.1.383) [72]. For each NR family, a boxplot was generated as an indication of the variability distribution within the whole family and the belonged members. Subsequently, for each receptor, the similarity matrix *S* was converted into a dissimilarity matrix *D* by using the transformation $d_{AB}=100-s_{AB}$. The resulting matrix contains all the pairwise comparisons of the *S value* in a reversed scale, where 0 indicates pairwise identity and 100 indicates pairwise maximum dissimilarity. Hierarchical clustering of matrix *D* was performed using an agglomerative approach and the complete linkage method from R and is presented in the heatmap plot. Finally, within each cluster, the medoid was detected as the one with the smallest sum of distances from the other elements.

## 5. Conclusions

In this paper, we presented an easy and fast methodology for the investigation of NR LBD structural flexibility, as a result of ligand-induced fit effects. The different binding site representations of each nuclear receptor were compared based on the conservation of residues at the pocket level and a flexibility profile for each receptor was outlined. Interestingly, while for some receptors, the binding site architecture was persistently conserved, for others, quite different conformations of the pocket were identified and should be considered when running structure-based drug design campaigns. Overall, the findings agree with the literature data and prove the reliability of the methodology. Moreover, the current work offers a global picture of the NR structural content in the PDB. Receptors with poor structural information, that is, a low number of solved structures, have been detected and are clearly identified in Table 1. The corresponding information drawn on flexibility must, thus, be considered with caution, because a possible low flexibility could be associated to the limited number of experimental structures. This could suggest that additional data on specific receptors, likely provided by computational analyses, could be useful when designing a structure-based drug design campaign. In cases in which a higher number of experimental structures has been solved, at a reasonably good resolution, the information related to the adaptability of NR LBD binding sites could be used with higher confidence. Indeed, we have provided a list of reference structures, representing different conformations of the binding site, which could be used in virtual screening campaigns for the identification and/or repurposing of new active molecules.

Overall, the methodology reported in this paper could be applied to any protein family retaining a conserved fold and mechanism of action, to study pocket dynamics and to identify representative structures from a pool of either experimentally or computationally derived conformations.

## Figures and Tables

**Table 1:** Structures and pockets analyzed for all NR sub-families.

Family	Sub-Family	Total LBD Structures	LBD Structures (R ≤ 2.5)	Final N° Liganded Pockets
NR1	CAR	2	/	/
	FXR	81	61	45
	LXRα	7	4	/
	LXRβ	13	9	7
	PPARα	18	15	9
	PPARδ	43	34	17
	PPARγ	204	156	40
	PXR	23	7	4
	RARα	6	4	4
	RARβ	7	5	4
	RARγ	11	11	10
	Rev-erba-A	1	/	/
	Rev-erba-B	4	4	/
	RORα	3	3	3
	RORβ *	/	/	/
	RORγ	88	62	46
	THRα	8	8	9
	THRβ	18	9	10
	VDR	49	39	39
NR2	COUP-TFI **	/	/	/
	COUP-TFII	1	1	/
	HNF4α	6	3	3
	HNF4γ	1	/	/
	PNR (E3)	1	/	/
	RXRα	80	38	38
	RXRβ	6	6	/
	RXRγ	1	1	/
	TLX (E1)	1	/	/
	TR2 (C1) *	/	/	/
	TR4 (C2)	1	/	/
NR3	AR	75	68	70
	ERα	274	227	148
	ER2β	32	27	27
	ERRα	4	4	/
	ERRγ	16	12	8
	GR	43	37	18
	MR	28	28	28
	PR	19	17	17
NR4	NGFI-B	15	12	5
	NOR1 *	/	/	/
	NURR1	4	4	3
NR5	LRH-1	16	14	14
	SF-1	4	4	4

The total number of analyzed LBD structures before and after filtering by resolution (R ≤ 2.5 Å) is reported. The final number of the analyzed liganded pockets, resulted from further filtering of the dataset according to the structural integrity criterion (see Materials and Methods), is reported in the last column. For receptors marked with a *, no structures have been deposited in the PDB. For receptors marked by **, only the DBD structure is available in the PDB.

**Table 2:** List of the representative structures for each nuclear receptor, extracted from the cluster analysis.

Family	Sub-Family	Representative Structures
NR1	FXR	5Q1H, 5ICK, 5Q0W
	LXRα	3IPU
	LXRβ	1PQ9, 3KFC
	PPARα	2P54, 5HYK
	PPARδ	3SP9, 5U3Z, 5U45
	PPARγ	3NOA, 2I4P, 1ZGY, 3TY0, 4E4Q, 6FZF
	PXR	1NRL, 5A86
	RARα	1DKF, 3KMR, 3KMZ
	RARβ	4JYI
	RARγ	1FCX
	RORα	1S0X
	RORγ	3KYT, 4WQP, 5X8Q, 6Q2W
	THRα	2H79
	THRβ	2J4A
	VDR	2HB8
NR2	HNF4α	3FS1
	RXRα	1FBY
NR3	AR	1T63, 3B67
	ERα	1L2I, 2AYR, 5DUH, 5FQP
	ER2β	1U9E, 2GIU
	ERRγ	2P7G, 2GPU
	GR	6EL7, 4CSJ
	MR	3VHU
	PR	1A28, 2OVH
NR4	NURR1	6DDA, 5Y41
	NGFI-B	4R38, 3V3Q
NR5	LRH-1	4PLE, 1YOK
	SF-1	4QJR

## Data Availability

Data are available upon request from the corresponding authors.

## Supplementary Materials

The following supporting information can be downloaded at: https://www.mdpi.com/article/10.3390/ijms23158732/s1.
