# Supplementary material for: Exploring Ligand Binding Domain Dynamics in the NRs Superfamily
Source: Int J Mol Sci. 2022 Aug 5;23(15):8732. doi: 10.3390/ijms23158732 (PMC9369052; doi:10.3390/ijms23158732)
Supplement: Supplementary file 1 [file ijms-23-08732-s001.zip › Supporting Information-revised2-final.pdf]

Supporting Information

Exploring Ligand Binding Domain variability in the NRs superfamily

Giulia D'Arrigo [1,§,#], Ida Autiero [2,3,#], Eleonora Gianquinto [1], Lydia Siragusa [2,4], Massimo Baroni [4], Gabriele Cruciani [5,6,*], Francesca Spyrakis [1,*]

[1] Department of Drug Science and Technology, University of Turin, Via Giuria 9, 10125, Turin, Italy; [2] Molecular Horizon Srl, Via Montelino 30, 06084 Bettona, Perugia, Italy; [3] National Research Council, Institute of Biostructures and Bioimaging, Naples, Italy; [4] Molecular Discovery Ltd., the Kinetic Centre, Theobald Street, Elstree Borehamwood, Hertfordshire WD6 4PJ, UK; [5] Department of Chemistry, Biology and Biotechnology, University of Perugia, Via Elce di Sotto 8, 06123, Perugia, Italy; [6] Consortium for Computational Molecular and Materials Sciences (CMS), Via Elce di Sotto 8, 06123, Perugia, Italy.

[§] Current address: Heidelberg Institute for Theoretical Studies (HITS), Schloss-Wolfsbrunnenweg 35, 69118 Heidelberg, Germany

[#] These authors contributed equally to the work

[*] Corresponding authors

gabri@chemiome.chm.unipg.it, francesca.spyrakis@unito.it

Table of content

Figure S1. Mouse-trap model……………………………………………………………...………………………S2

Figure S2. Heatmaps for the remaining receptors of NR1 family…………………………………………….....S3

Figure S3. Heatmaps for the remaining receptors of NR3 family………………………………………………S4

Figure S4. Heatmaps for the remaining receptors of NR2 family…………………………………………….....S5

Figure S5. Superposition of the four ERα medoids…………………………………...……………………..S6

Figure S6. PCA score plot calculated for GR pocket MIFs……………………………………………………S7

Details on GRID algorithm and pocket comparison performed by FLAP………………………………..….S8

Figure S1.

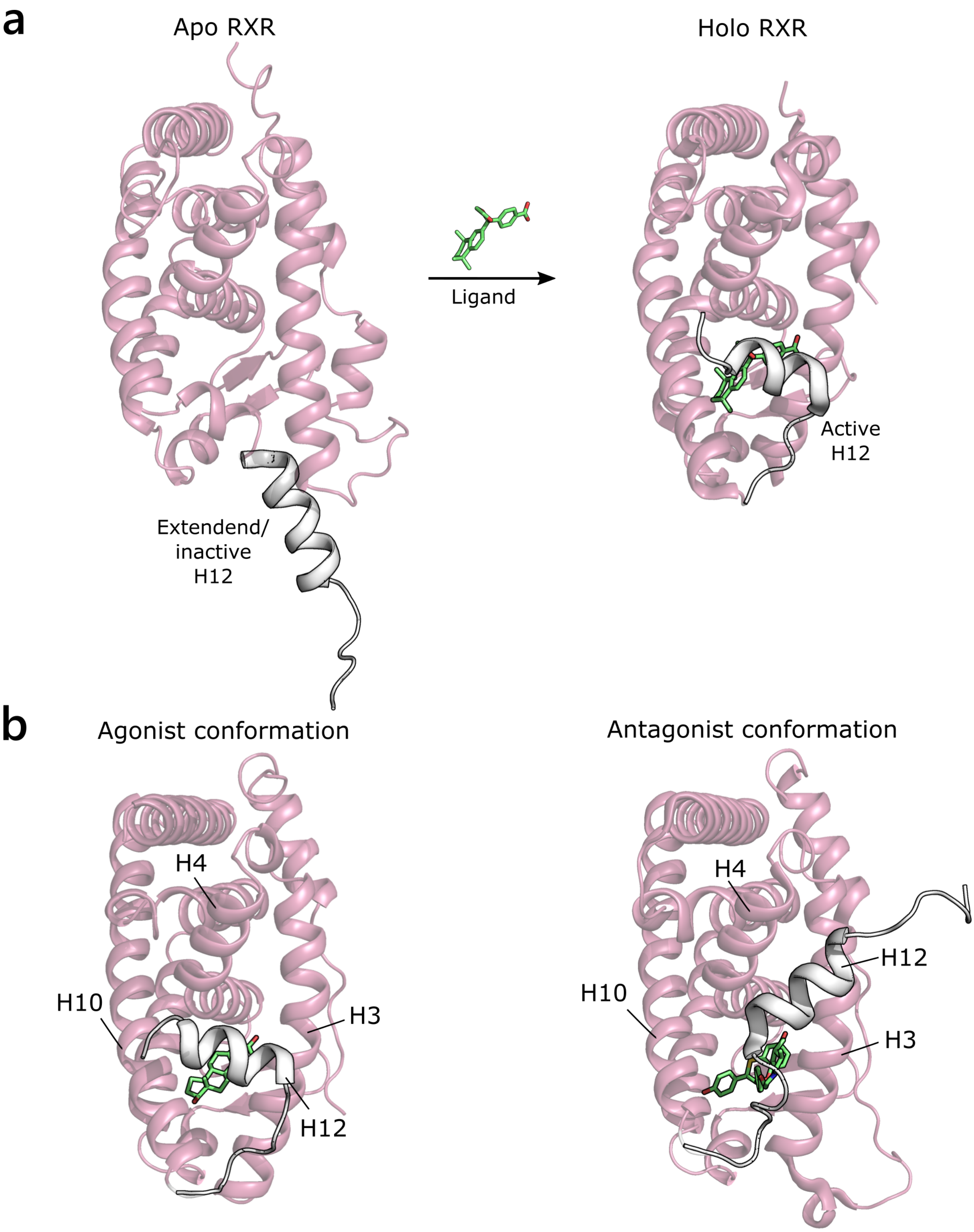

Figure S1. a. Mouse trap model: comparison of the apo and the holo structure of RXR showing a massive rearrangement of H12 upon ligand binding (PDBs: 1LBD apo, 1MVC holo). **b.** Different H12 conformation as result of the ligand-induced fit upon agonist (on the right) and antagonist (on the left) binding in ERa. (PDBs: 1ERE agonist, 1XP1 antagonist).

Figure S2.

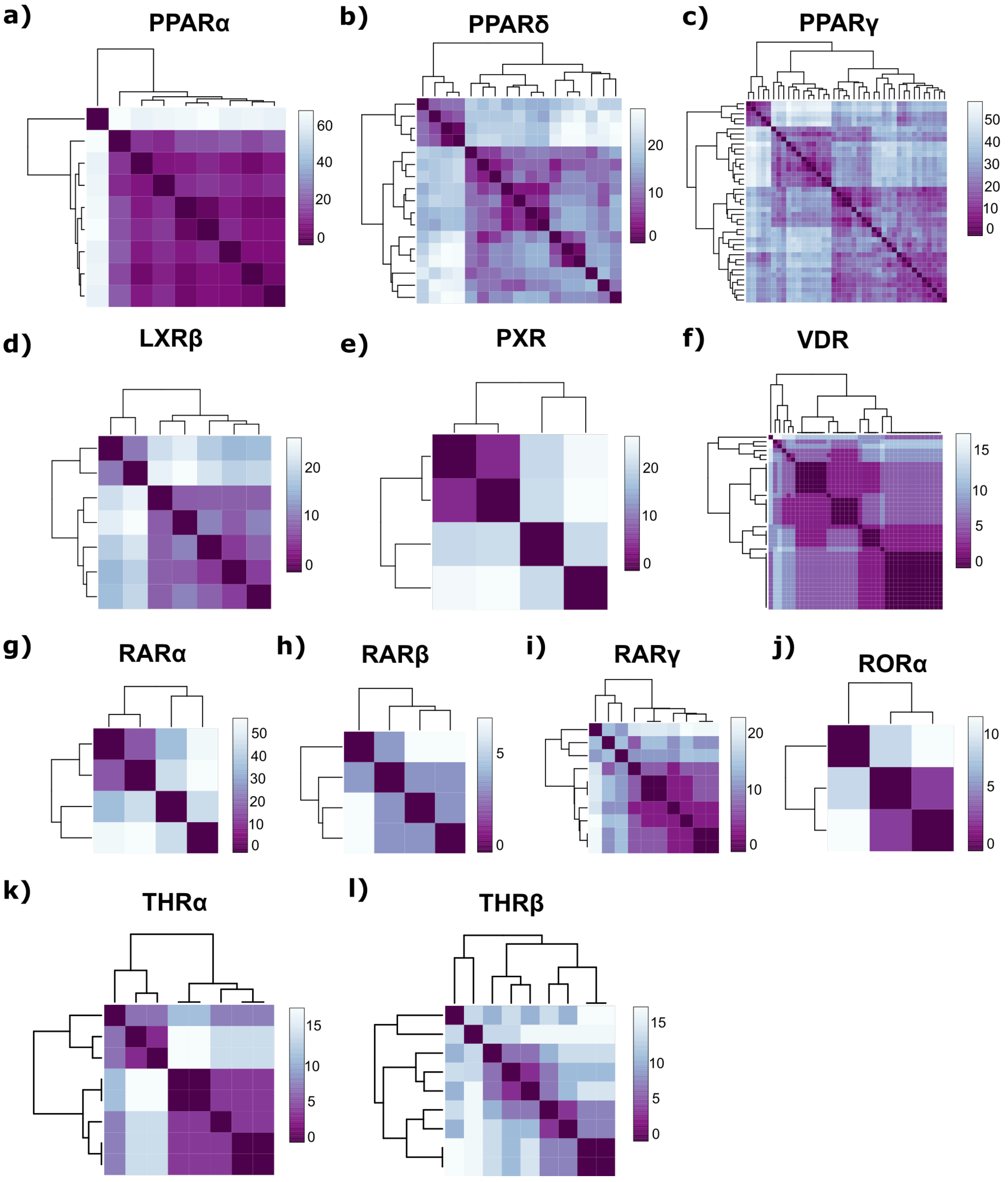

Figure S2. Heatmaps for the remaining receptors of NR1 family.

Figure S3.

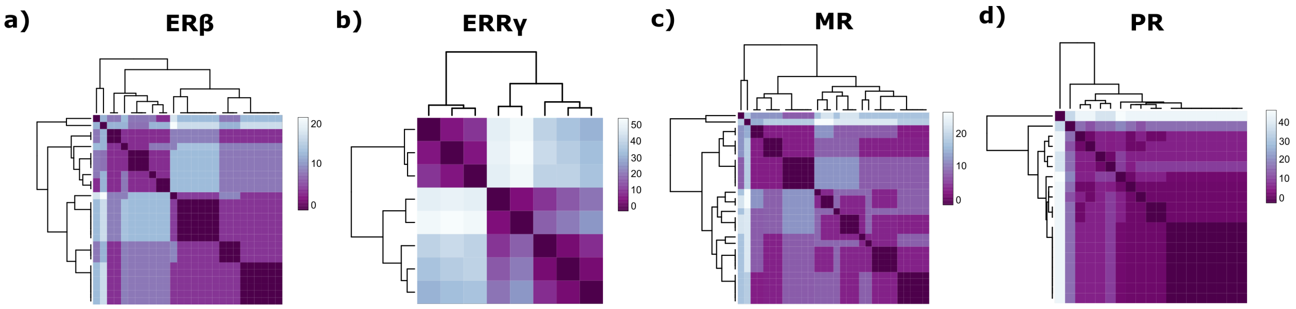

Figure S3. Heatmaps for the remaining receptors of NR3 family.

Figure S4.

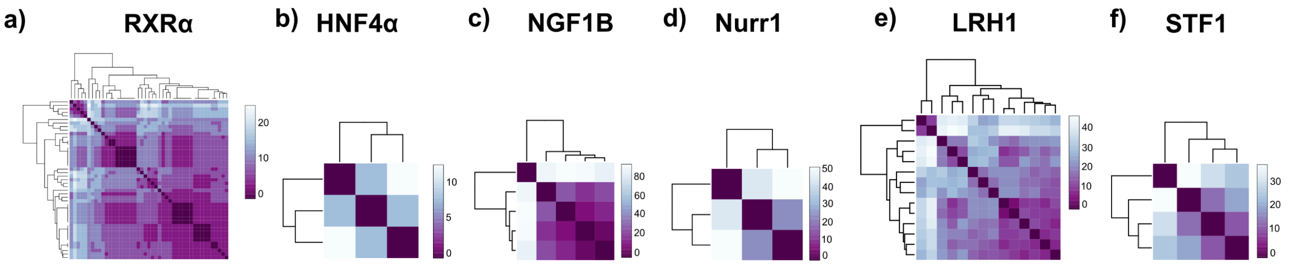

Figure S4. Heatmaps for the remaining receptors of NR2 family.

Figure S5.

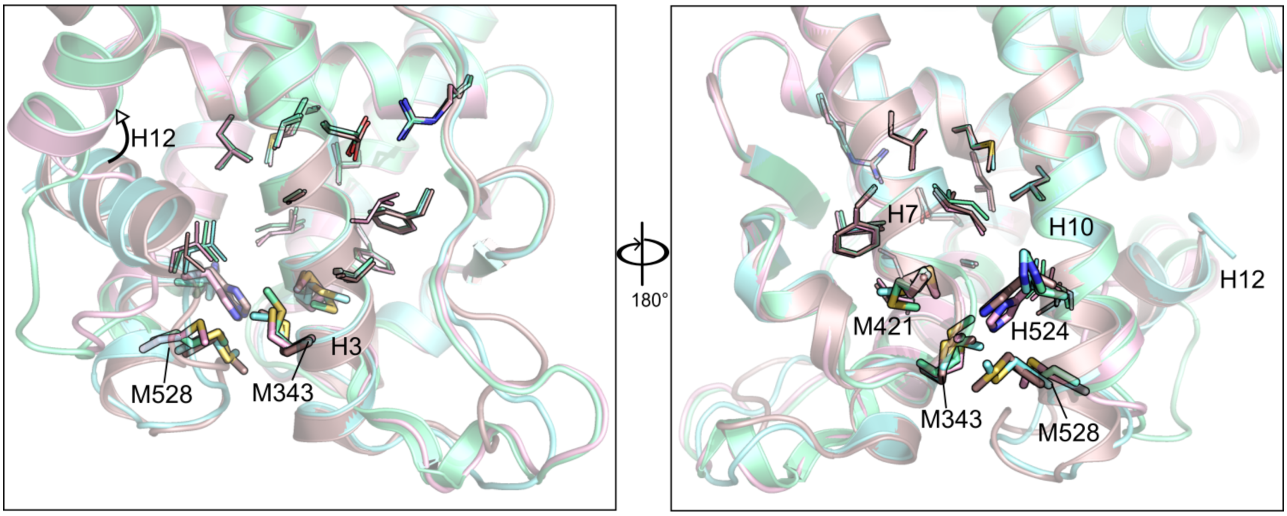

Figure S5. Superposition of the four ERa medoids. Each medoid is shown as cartoon and colored according to the clustering color code. Binding site residues are shown as lines while the four most flexible residues (Met343, Met421, His524 and Met528) are represented as sticks and labelled. On the right, H12 transition from the agonist to the antagonist conformation is highlighted.

Figure S6.

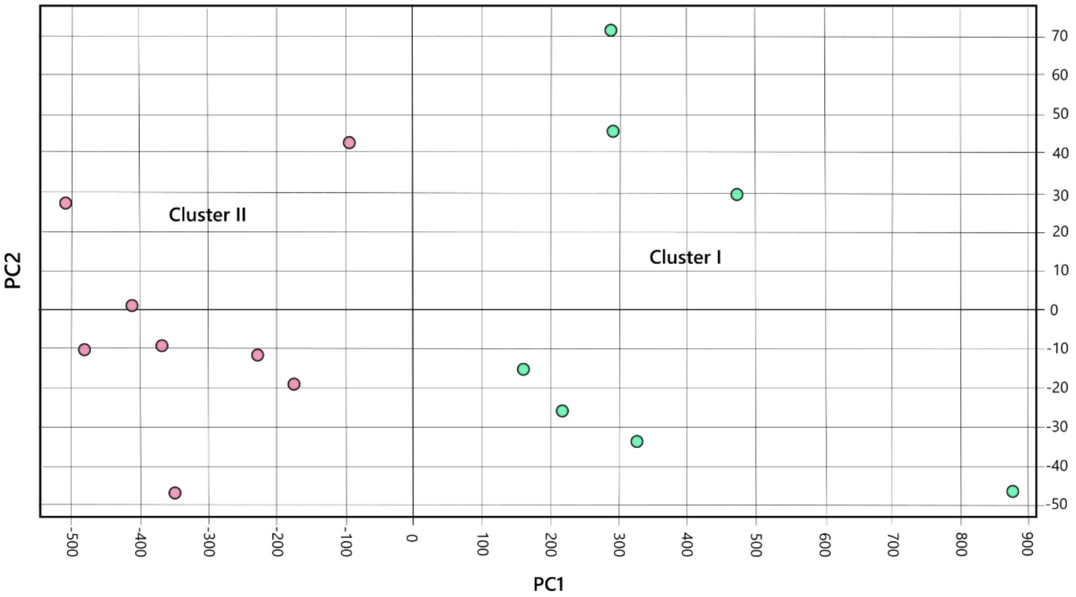

Figure S6. PCA score plot calculated for GR pocket MIFs. The two groups of pockets are differently coloured, cluster I in green and cluster II in pink.

Details on GRID algorithm and pocket comparison performed by FLAP

Cavities are processed by the GRID force field [71] to evaluate the type, strength and direction of the interactions that a cavity is capable of making. The GRID probes H, DRY, O, and N1 are used to compute the shape, the hydrophobic interactions, the H-bond acceptor interactions and the H-bond donor interactions respectively for each cavity considered in the analysis. The energy of the interaction of the N1 and O probes includes implicitly information about the charge contribution. The MIFs that are generated whilst being very informative, have the disadvantage of being difficult to handle from a computational perspective compared with fingerprint approaches. Thus, the FLAP algorithm first reduces the complexity of the MIFs by selecting a number of representative points using a weighted energy based and space-coverage function. Basically, cavity is divided in distinct regions that are related to the single residue responsible of the best optimal interaction with the probe. For each region, it is then assigned a fraction of representative points (in respect of the overall amount of points computed at the beginning of the GRID run) that are proportional to the strength of the interaction energy and characteristics of the associated residue. FLAP then generates all possible combinations of four of the representative points, with each combination termed a quadruplet. For each quadruplet the four points together with the six distances are stored along with the volume of the quadruplet which retains information about chirality. This information about the spatial arrangement of interactions is one of the key differences between 2D and 3D similarity methods. All quadruplets generated for a cavity are represented as a bitstring that constitutes the “Common Reference Framework.” Given a dataset of cavities, all such common reference frameworks are stored into a database to perform a comparison with one or more template structures to calculate the degree of similarity between them [21].
